# How Can Debiasing Research Aid Efforts to Reduce Discrimination?

**DOI:** 10.1177/10888683241244829

**Published:** 2024-04-22

**Authors:** Jordan Axt, Jeffrey To

**Affiliations:** 1 McGill University, Montreal, Quebec, Canada

**Keywords:** discrimination, debiasing, biases, judgment and decision-making, intergroup relations

## Abstract

Understanding and reducing intergroup discrimination is at the forefront of psychological research. However, efforts to find flexible, scalable, and durable interventions to reduce discrimination have produced only mixed results. In this review, we highlight one potential avenue for developing new strategies for addressing discrimination: adapting prior research on *debiasing*—the process of lessening bias in judgment errors (e.g., motivated reasoning, overconfidence, and the anchoring heuristic). We first introduce a taxonomy for understanding intervention strategies that are common in the debiasing literature, then highlight existing approaches that have already proven successful for decreasing intergroup discrimination. Finally, we draw attention to promising debiasing interventions that have not yet been applied to the context of discrimination. A greater understanding of prior efforts to mitigate judgment biases more generally can expand efforts to reduce discrimination.

**Public Abstract:**

Scientists studying intergroup biases are often concerned with lessening discrimination (unequal treatment of one social group versus another), but many interventions for reducing such biased behavior have weak or limited evidence. In this review article, we argue one productive avenue for reducing discrimination comes from adapting interventions in a separate field—judgment and decision-making—that has historically studied “debiasing”: the ways people can lessen the unwanted influence of irrelevant information on decision-making. While debiasing research shares several commonalities with research on reducing intergroup discrimination, many debiasing interventions have relied on methods that differ from those deployed in the intergroup bias literature. We review several instances where debiasing principles have been successfully applied toward reducing intergroup biases in behavior and introduce other debiasing techniques that may be well-suited for future efforts in lessening discrimination.

“Bias” is one of the most impactful words in psychological research. Many studies have sought to identify how bias develops, who is most likely to show bias, and how bias can be reduced. But in the decades that bias has remained a focal topic among psychologists, a general split has emerged in what constitutes a biased attitude or judgment. For researchers focused on the psychology behind debiasing judgment, bias refers to contexts where irrelevant or nondiagnostic information exerts an undue or irrational influence on our perceptions, beliefs, or behaviors. From this perspective, relatively asocial phenomena can be considered forms of bias. For instance, hindsight bias (i.e., the “I knew it all along” effect) is partly driven by selectively recalling information consistent with what people now know to be true (Roese & Vohs, 2012); in other words, an overreliance on certain information in memory biases our present beliefs. In another example, confirmation bias can arise from the imperfect interpretation of available evidence (Nickerson, 1998), giving more weight to outcomes that support our pre-existing opinions, which in turn biases the beliefs we form.

Quantifying the number of such biases in human cognition is not easy; Wikipedia, for example, lists more than 200 different “cognitive biases” (“List of Cognitive Biases,” 2022). While some perspectives argue that these judgment biases do not necessarily reveal irrational judgment but rather responses to shifting needs and cognitive resources (e.g., Gigerenzer & Goldstein, 1996), the dominant interpretation of such biases remains that they reflect a deviation from purely rational judgment that arises from the flawed processing of available information (e.g., Kahneman & Tversky, 1996). The present work focuses on prior investigations of these judgment biases, specifically reviewing the various strategies that have been deployed to reduce their strength and impact (i.e., “debiasing”). We then explore how these strategies may or may not be productively applied to a distinct but related field of social psychological research: intergroup biases in judgment and behavior.

Unlike researchers focused on decision-making errors, researchers in intergroup relations see the word “bias” as having a more intrinsically social component. Here, bias largely refers to preferences in attitudes, beliefs, or behaviors for one social group over another. To that end, a recent Google Scholar search for “intergroup bias” revealed over 24,000 articles. This view of “bias” refers more to the tendency to have more favorable evaluations or treatment of one social group relative to another, typically emerging as favoritism for one’s own group (Hewstone et al., 2002). Intergroup bias is then closely related to the phenomenon of prejudice (affective associations about groups) and discrimination (behavioral differences in treatment based on group membership; Fiske, 1998).

Broadly, these two conceptions of bias—one more focused on relatively asocial judgment heuristics and the other on intergroup preferences—have obvious similarities. Both approaches assume that evaluation, perception, and behavior can be irrationally or overly influenced by one’s larger goals, such as the desire to appear correct in the case of confirmation bias (Nickerson, 1998) or the motivation to privilege one’s ingroup as a means of boosting self-esteem (Brewer, 1979). Indeed, seminal papers on bias and debiasing (i.e., removing bias) have drawn connections between the two perspectives. For instance, Wilson and Brekke’s (1994) model of “mental contamination”—where unwanted attitudes or behavior emerge due to unregulated, automatic mental processes—begins with two examples of mental contamination that mirror these two conceptions of bias. In the first example, a consumer decides to purchase a product based on the framing used in an advertisement, and in the second example, a teacher unknowingly favors a more physically attractive student. From this perspective, being swayed by the one-sided information presented in an advertisement and discriminating in favor of physically attractive people are both instances of biased behavior.

Conceptually, too, the two uses of “bias” share clear parallels. In one example, work in debiasing judgment argues that biased behavior partly emerges because “people often form quick and intuitive judgments based on limited information. . . which may be incomplete or ambiguous” (Soll et al., 2015), a framework that is highly consistent with prominent models of intergroup biases, which argue that, in many cases, prejudice emerges through the quick and faulty encoding of available information (e.g., Fyock & Stangor, 1994; Taylor et al., 1978). For instance, the phenomenon of an illusory correlation (Hamilton & Rose, 1980), in which information encountered less frequently is prioritized in attention and memory, can lead to intergroup prejudices (e.g., J. W. Sherman et al., 2009).

Yet, there are also notable differences between these two conceptions of bias. For instance, intergroup biases are typically embedded and informed by cultural conceptions of group status or value (e.g., Axt et al., 2014) and, as a result, seem to be much more culturally dependent than other types of judgment biases (e.g., Ruggeri et al., 2022). In addition, while phenomena like confirmation or hindsight bias are believed to reveal forms of irrational and erroneous thinking, intergroup relations researchers are more divided on whether intergroup bias must necessarily reflect irrational attitudes and behavior; that is, some types of intergroup bias may be justifiable or even expected (e.g., biases against drunk drivers; Crandall & Eshleman, 2003). While such differences are worth remembering, this review focuses on the empirical and conceptual similarities between these two forms of bias and how we can apply this understanding to reducing bias in intergroup behavior.

Despite any potential connections between these two literatures on bias, they appear to rarely speak to one another. For instance, for three prominent “debiasing” reviews (Fischhoff, 1982; Larrick, 2004; Milkman et al., 2009), only 3% of 2022 papers citing these articles came from research on intergroup prejudice or discrimination.^1^ Similarly, of three prominent reviews concerning intergroup biases (Hewstone et al., 2002; Paluck et al., 2021; Paluck & Green, 2009), no papers published in 2022 citing this work came from the fields of judgment and decision-making. The lack of dialogue between these two perspectives may not only impede theoretical understanding of the broader structure behind biased attitudes and behavior but can also obstruct more practical efforts for identifying effective approaches to reduce bias.

In this review, we look to bridge these two literatures by exploring how efforts to lessen intergroup discrimination can be informed by studies in debiasing. Specifically, we identify novel interventions in work on debiasing that could supplement the relatively small number of successful strategies that have arisen in the intergroup bias literature (e.g., Paluck et al., 2021)—particularly considering that many interventions have struggled to prove to be flexible, scalable, and capable of creating lasting change (e.g., E. H. Chang et al., 2019; Hsieh et al., 2022; Lai et al., 2016). A deeper understanding of debiasing research may in turn propel novel insights and efforts for reducing intergroup biases in behavior.

First, we summarize several prominent approaches in the debiasing literature. We then use this framework to (a) discuss why certain interventions are likely ineffective in the domain of intergroup biases in behavior, (b) highlight recent instances where researchers successfully used debiasing approaches to reduce discrimination, and (c) outline several promising debiasing strategies yet to be widely applied to intergroup contexts. In all, this paper seeks to identify useful methods of reducing judgment biases in the debiasing literature and use these results to motivate and inform future research in intergroup bias.

It should be noted that most of the cited studies and future recommendations focus on reducing discrimination (i.e., intergroup biases in behavior) rather than prejudice (i.e., intergroup biases in attitudes). This is due to several reasons. For one, much of the reviewed research in debiasing uses behavioral outcomes, which make for a more natural application to questions of discrimination. Moreover, the roots of intergroup prejudice are multiple (e.g., Rudman et al., 2007), and people are less likely to control these affective responses than they can control their behavior. Thus, an effective approach for reducing intergroup disparities may rely less on changing people’s attitudes and more on making sure these attitudes do not transfer to behavior.

This distinction between shifting attitudes and shifting behavior is shown clearly in a classic study on ignoring impermissible court testimony during a mock jury task (Schul & Manzury, 1990). Participants were presented with unflattering information about the defendant in an assault case, but were told to ignore this information because the evidence was considered inadmissible. Results found that the presence of the unflattering information impacted *impressions* of the defendant (i.e., the defendant was viewed as more aggressive), but did not alter their final *verdicts* (i.e., the experimental condition was no more likely to render a guilty verdict than a control condition that never saw the inadmissible evidence). To the extent that intergroup behaviors are also likely to be under more conscious control than intergroup attitudes, interventions will be more effective on discriminatory behaviors than prejudices.

Furthermore, our review largely focuses on changing forms of *unintended* discrimination (Bertrand et al., 2005), where behaviors suggest preferences that would not align with an individual’s conscious goals. One reason for this focus is that these unintended forms of discrimination have the closest parallels to the judgment biases that are mostly investigated in the debiasing literature, where such biases are typically viewed as irrational or undesired by individual decision-makers (Hilbert, 2012). In this sense, both traditional judgment biases and unintended discrimination are a form of “mental contamination” (Wilson & Brekke, 1994), where judgments or behaviors are impacted by mental processes that are outside of conscious awareness or conscious control. However, while the broad similarities across the two forms of biased judgment allow for the potential for highlighting shared mechanisms, there are several weaknesses to this approach. Specifically, this perspective may not give proper weight to the role of pre-existing attitudes toward or experiences with members of other groups (e.g., Rudman, 2004), nor does it directly incorporate historical or systemic factors that can shape intergroup behavior (e.g., Skinner-Dorkenoo et al., 2023).

A second reason for our focus on behavior is that past research has identified many instances where intergroup behaviors can change *without* corresponding attitude change (e.g., Mousa, 2020; Scacco & Warren, 2018), illustrating that attitude change is not a requirement for reducing discriminatory behavior (see Brauer, 2024 for review). A final reason is that most people self-report high levels of motivation to be non-prejudiced in many social domains (Bamberg & Verkuyten, 2022), meaning that in many cases, when discrimination emerges, it is due to the influence of psychological processes that are not personally endorsed.

Of course, this is not a full description of all forms of intergroup bias, and this review cannot speak to efforts for limiting several prominent forms of prejudice or discrimination. For one, social group membership is crucial for satisfying basic psychological needs (e.g., Hornsey & Jetten, 2004). Many forms of discrimination may then manifest as promoting or protecting one’s ingroup, and this focus on bolstering one’s ingroup hinders recognition that such behaviors are discriminatory toward outgroup members (e.g., Greenwald & Pettigrew, 2014). In addition, a non-trivial portion of the population has a conscious desire to express prejudice in their beliefs and behaviors (e.g., Forscher et al., 2015). Addressing forms of discrimination rooted in ingroup favoritism—in addition to more deeply held forms of intergroup bias—is a worthy topic of research (e.g., Bar-Tal & Hameiri, 2020) but is outside the scope of this review.

In addition, the research reviewed here comes primarily from Western cultures, though we have sought to cite evidence from other world regions when possible. The non-representative nature of the available evidence has clear implications for generalizability. Though several judgment biases appear to be robust across many cultures (e.g., Klein et al., 2018; Ruggeri et al., 2022), other evidence suggests that one’s cultural environment can influence factors related to decision-making and intergroup beliefs or behavior (e.g., Lu et al., 2020; Mezulis et al., 2004). Research in this topic will benefit greatly from additional applications of the interventions reviewed here to better understand when effects do versus do not generalize across cultures and samples. Finally, this review does not provide an exhaustive account of all potential avenues for lessening discrimination, as there are many other promising approaches for reducing discriminatory judgment (e.g., correcting meta-perceptions; Lees & Cikara, 2020). Rather, this work focuses more narrowly on reviewing how several promising strategies from the debiasing literature could be adapted to the study of bias in intergroup behavior.

## Approaches to Debiasing

Fischhoff (1977) was the first to use the word “debias” in the psychological literature. Since then, several reviews have emerged on common debiasing strategies (Arkes, 1991; Fischhoff, 1982; Larrick, 2004; Soll et al., 2015; Wilson et al., 2002). These reviews highlight three common strategies for debiasing judgment and behavior: (a) changing an individual’s ability or motivation to combat bias; (b) changing the processing strategy used to navigate a biasing context; (c) changing how judgments are structured to naturally elicit less-biased decision-making. Below, we provide more detail and review foundational studies that illustrate the effectiveness of these strategies. Later, we discuss how they can be applied to reducing intergroup discrimination. See Table 1 for an overview of the various debiasing approaches we review, the suggested mechanisms behind those debiasing approaches, instances of similar approaches used to address intergroup discrimination (if available), and an example of how such strategies could be applied to real-world evaluation contexts.

**Table 1.** Overview of Debiasing Approaches and Applications Within Intergroup Contexts.

Approach	Debiasing example	Suggested mechanism	Intergroup literature example	Sample application in intergroup contexts
***Change ability***	Training (Lehman & Nisbett, 1990)	Greater decision-making skill and capacity	Practice with feedback reduced reduce attractiveness-based discrimination (Roy et al., 2024)	Where possible, training evaluators on past applicants and providing feedback on correct/incorrect decisions; training employees on one form of bias (e.g., confirmation bias) in hopes that training transfers to other forms of intergroup bias.
***Change motivation***	Financial incentives (Ariely, Bracha, & Meier, 2009)	Increased effort	Financial incentives had no impact on attractiveness-based discrimination (Axt et al., 2024)	Not recommended in intergroup contexts
	Accountability (Lerner & Tetlock, 1999)	Heightened feelings of self-criticism	Feelings of accountability reduced ingroup favoritism in an allocation task (Dobbs & Crano, 2001)	Warning leasing officers that they will need to justify their evaluation of each housing applicant to other leasing officers.
***Change processing strategy***	Consider the opposite/alternative (Lord et al., 1984)	Increased uncertainty; Reduced use of simulation heuristic	Not yet applied	During parole hearings, ask board members to articulate reasons why the opposite decision may be appropriate (e.g., why a person should versus should not receive parole).
	Linear models and algorithmic decision-making (Samson & Thomas, 1987)	Reduced reliance on noisy or undiagnostic criteria	Not yet applied	Using a decision rule that both promotes the use of decision-relevant criteria but is also aligned with individual/organizational values in terms of representation and diversity.
***Change how decisions are presented***	Accentuating specific information (Sedlmeier & Gigerenzer, 2001)	More intuitive information formats promote more logical reasoning	Not yet applied	Exploring whether decision biases are lessened when college applicants’ information is presented in a variety of formats (e.g., numerical versus percentile presentation of high school class rank).
	Altering choice framing (Rothman & Salovey, 1997)	Frames (e.g., gains vs. losses) increase promotion versus prevention thinking	Not yet applied	Emphasizing strengths (instead of weaknesses) of applicants when deciding which employee should receive a promotion.
***Change how decisions are structured***	Pre-commitment (Thaler & Benartzi, 2004)	Heightened consistency between values and behaviors	Pre-commitment to criteria eliminated gender bias in a hiring task (Uhlmann & Cohen, 2005)	Pre-committing to *diagnostic* criteria when formulating insurance rate estimates (in a way that does not exacerbate existing intergroup disparities).
	Judgment decoys (Ariely & Wallsten, 1995)	Superior options are easier to identify	Decoys reduced gender-based discrimination in a selection task (L. W. Chang & Cikara, 2018)	When deciding who to give a financial loan, ensure that financial profiles of dominant groups with poorer financial histories are included to emphasize the strengths of financial profiles of minorities.
	Changing defaults (van Kleef et al., 2018)	Defaults suggest preferred action; inertia increases reliance on default option	Opt-out framing attenuated gender-based discrimination (He et al., 2021)	During selection for leadership programs, making self-nomination the default; have marginalized groups automatically enroll in mentorship programs; and place minority students as scholarship-eligible by default.

### Changing Ability or Motivation

The first approach to debiasing judgment focuses on altering participants’ ability or motivation to parse biasing information. Motivation and ability are two prominent moderators of many psychological phenomena (e.g., Fazio, 1990; Wegener & Petty, 1997), and biased judgment appears to be no exception.

#### Changing Ability

One strategy for debiasing is to increase people’s level of expertise in the judgment domain (i.e., to undergo training). In particular, training studies look to provide people with a greater ability to overcome a specific decision or judgment known to create bias, and then explore whether this training carries over to similar instances of that same judgment or generalizes to conceptually related judgments. Support for this approach has emerged in both short-term laboratory data as well as in longitudinal studies. For example, Lehman and Nisbett (1990) found that students majoring in the social sciences showed improvements in statistical reasoning (e.g., more sensitivity to the law of large numbers) when tested in their last versus first year of college. Notably, these improvements over time were larger for students in social science than those in the humanities or natural sciences, suggesting that the courses required to complete a social science degree equipped students with better reasoning abilities. Similar, though less conclusive, evidence of the benefits of expertise comes from work on weather forecasters (Murphy & Winkler, 1977), who showed remarkable accuracy in predictions like rain probabilities. One means by which forecasters develop expertise, and thereby a less-biased judgment, is through continual, unambiguous feedback: when weather forecasters urge us to ditch our umbrellas on what turns out to be a rainy day, they receive a clear signal from their viewers that they must revise their evaluation strategies.

Expertise in specific types of judgment can also be generated more quickly, such as within a single study session. For instance, participants showed better performance on a logical reasoning task after a brief form of training in deductive reasoning (Cheng et al., 1986). In more recent work, an interactive training reduced levels of confirmation bias in a novel judgment context by (a) eliciting a form of confirmation bias, (b) providing feedback on the level of bias exhibited, and (c) showing videos of experts explaining the nature of the bias (Morewedge et al., 2015). A follow-up study in the same paper found that the method extends to reductions in other judgment biases, such as the anchoring and representativeness heuristics. This work has since been conceptually replicated (Sellier et al., 2019), as business school students who received the Morewedge et al. (2015) training showed superior performance when navigating a case study that often elicits confirmation bias. Separate work has found that playing a game known to evoke confirmation bias (and receiving feedback about one’s own level of bias on the task) produced long-term changes in biases like the fundamental attribution error (Dunbar et al., 2017). More recent efforts have shown positive effects (at least in the short term) of training for reducing the base-rate fallacy in judgment among a sample of Himba tribe members living in Namibia (Boissin et al., 2024).

While these studies demonstrate the possible benefits of training in reducing biased judgment, concerns remain over issues like how targeted training needs to be and whether training effects can transfer to novel contexts. For instance, the previously mentioned study on training in logical reasoning (Cheng et al., 1986) was only effective when participants received lessons in *both* abstract principles of reasoning as well as specific examples to practice; neither component changed reasoning performance on its own. Regarding knowledge transfer, a separate study of weather forecasters (Wagenaar & Keren, 1986) found that forecasters were *more* likely than university students to show an overconfidence bias when completing a set of general knowledge questions. Weather professionals would often assign 100% confidence in their answers to a general knowledge question, a behavior that would be quite rare in their domain of expertise. In addition, even highly relevant expertise does not always translate into reduced bias; in one example, professional philosophers were just as susceptible to order effects as nonexperts when evaluating the morality of others’ behavior (Schwitzgebel & Cushman, 2012). This work demonstrates that while some forms of training have produced durable and generalizable reductions in biased behavior, a larger review of the literature suggests more equivocal results.

Given these mixed findings, it is no surprise that identifying the conditions needed for training to be effective is a common discussion in the debiasing literature. Indeed, Fischhoff (1982) listed the following as necessary components of a training or education program to reduce biased judgment: (a) abundant practice, (b) clear criterion and outcome measures, (c) task-specific reinforcement (i.e., feedback or reward), and (d) admission of the need for learning. However, many contexts cannot easily accommodate these conditions; for example, hiring managers often lack objective indicators of what makes a successful employee (meaning there are no clear criterion measures) or cannot know how unselected applicants would have fared if given the opportunity (meaning they lack comprehensive feedback). As a result, it is perhaps unsurprising that the evidence behind research on expertise and training as methods for debiasing is ambiguous, and later, we review the procedural factors that we believe will maximize the chances that similar approaches are effective in reducing discrimination.

#### Changing Motivation

A related approach concerns changing an individual’s motivation to be unbiased. In these cases, it is presumed that an unbiased or less-biased behavior is possible by encouraging deep or deliberative thinking. A review of these motivational tactics suggests that some strategies are more effective than others. For instance, financial incentives are a commonly used method to raise motivation (Giles et al., 2014; Maki et al., 2016), but the evidence that such incentives can debias judgment is weak (Soll et al., 2015). Though incentives have shown clear effectiveness in contexts like weight loss (e.g., John et al., 2011) and exercise (Jansons et al., 2016), they have proven less successful in contexts that require more cognitively complex behavior (Bonner et al., 2000). In some cases, offering rewards can even worsen judgment or behavior (e.g., Ariely, Bracha, & Meier, 2009). In one example, a field study in India (Sudarshan, 2017) found offering financial incentives for reducing electricity consumption erased any gains that emerged from an intervention targeting social norms (i.e., how customers’ usage fared relative to their neighbors). In short, financial incentives may be productive in areas where the correct behavior is relatively clear but people are prone to low effort (e.g., Allcott et al., 2020), but they can be ineffective or detrimental in contexts where either the correct response or strategy is unclear or people already feel motivated to perform well (e.g., Ariely, Gneezy, et al., 2009).

Another strategy for increasing motivation relies on invoking feelings of accountability, where individuals must (or expect to) justify their behavior to others. Accountability has been shown to reduce numerous biases in judgment (Lerner & Tetlock, 1999), and one potential mechanism for its effectiveness is heightening feelings of self-criticism, which in turn reduces heuristic or low-effort thinking. In one example, participants performed a group decision-making task that involved selecting an employee to work at a home for adolescents who had been convicted of a crime (Huber & Seiser, 2001). Applicants were presented with 16 attributes that varied from less relevant (being a smoker) to more relevant (years of experience in social work). Relative to a condition that received no further information, groups that completed the task following an accountability intervention (i.e., believing that they would need to write a letter outlining their judgment process) ended up considering a greater number of attributes when evaluating applicants and considered these attributes more consistently during the evaluation process. More recently, heightening feelings of process accountability (i.e., that one must justify how they navigated a decision) led to a more thorough search for decision-relevant information and superior overall judgments when making financial decisions (Dalla Via et al., 2019).

To be clear, this is not an exhaustive list of strategies to increase motivation, and related literature suggests other possible routes. For instance, education researchers have found that invoking feelings of self-efficacy (i.e., perceptions that one can accomplish the task at hand) can result in greater persistence in academic-related tasks (e.g., Schunk, 1995), and heightening self-efficacy could in turn motivate people to make more effortful and unbiased decisions. Another option comes from the intergroup literature, where asking people to reflect on their core values can enhance motivations to be egalitarian (D. K. Sherman et al., 2017). From this perspective, reflecting on core values (such as one’s internal motivation to be unprejudiced; Lacosse & Plant, 2020) could be another route for lessening unfair or discriminatory behavior. In all, shifting motivation remains a promising strategy for reducing some forms of biased judgment. Even in contexts where people are motivated to be unbiased, such interventions can still guide participants to deliberate or invest more in judgment, leading to less bias in decision-making.

### Change the Processing Strategy

Interventions that focus on changing ability or motivation seek to equip people with greater judgment prowess or an increased desire to be unbiased, meaning these approaches look to alter an aspect of the individual that is then carried over into a novel context. In contrast, interventions looking to change the processing strategy are more localized and provide tactics that can be applied directly to the judgment at hand. Two popular examples of this approach are interventions that prompt people to “consider the opposite/alternative” or provide linear models that can be used to simplify judgment.

#### Consider the Opposite and Consider the Alternative

One example of debiasing through changing the processing strategy is by guiding people to consider multiple alternative outcomes, or to “consider the opposite” (Lord et al., 1984). There are multiple accounts for why considering counter-explanations could debias judgment. One mechanism is that the procedure introduces an “averaging rule”: when people are confronted with an alternative or opposing possibility for an outcome, rather than choose one or the other they split the difference, thereby landing on a relatively moderate (or less-biased) judgment (Van de Calseyde & Efendić, 2022). Similarly, another potential mechanism is that generating alternative explanations increases feelings of uncertainty, which prompts participants to endorse the “middle” outcome (or indifference point) to reflect this uncertainty (Hirt & Markman, 1995). Finally, considering the opposite could work through a simulation heuristic: after considering multiple possible outcomes (alternative or opposing), participants weigh the likelihood of each outcome based on how easily they are generated—the outcomes most easily generated are also judged as more likely and the outcomes less easily generated are judged as less likely (Kahneman & Tversky, 1982).

Regardless of the specific mechanism behind the results, considering the opposite or the alternative has been a widely successful debiasing strategy. In one application of this approach, participants’ hindsight bias—in this case, their belief that a scientific finding would replicate across new data collections—was lessened in a “foresight” condition that prompted participants to envision and explain both possible outcomes (i.e., both a successful and a failed replication; Slovic & Fischhoff, 1977). In other words, merely entertaining a different outcome was enough to reduce hindsight bias.

Similar “consider the opposite” strategies have been effective in other forms of biased judgment. One study reduced the overconfidence bias—here, when estimates of accuracy for general trivia knowledge exceeded actual accuracy—by asking people to list the potential reasons why their answers may have been wrong (Koriat et al., 1980). In another instance, the biased evaluation of a scientific study that was or was not consistent with one’s pre-existing beliefs—a form of confirmation bias—was weakened following a prompt that asked participants to consider whether they would have had the same opinion of the study’s quality if it had reached the *opposite* conclusion (Lord et al., 1984). More recently, Nagtegaal et al. (2020) applied the “consider the opposite” strategy to another prominent bias, as participants showed weaker levels of an anchoring bias (Tversky & Kahneman, 1974) when asked to consider why their initial anchor could have been inappropriate. Related work has sought to leverage “the crowd within.” For instance, participants who had to complete several estimation tasks (e.g., guess the weight of a grand piano) were more accurate when having to average between (a) their initial guess and (b) the guess of someone whom they imagined as disagreeing with them (Van de Calseyde & Efendić, 2022). This approach was found to be more effective than averaging with the imagined response of someone with whom the participant agreed (see Fujisaki et al., 2023 for a successful extension of this approach in a sample of Japanese participants). Across a range of contexts, prompting participants to consider opposing information has been shown to reduce biases in judgment.

A related strategy is to “consider the alternative.” Where considering the opposite involves imagining a contradictory scenario, consider the alternative asks people to consider any plausible outcome of an event. In one study using this approach, participants were asked to estimate how strongly personality traits like riskiness and conservatism predict success as a firefighter (Hirt & Markman, 1995). After reviewing information that showed no evidence of such an association, participants were then asked to explain why a possible association between these traits and success *could* exist. In one condition, they were asked to explain a single type of association (e.g., a negative correlation between riskiness and successful firefighting), and in a second condition, they were asked to explain this association and then a second, alternative association (e.g., also explaining why there could be no correlation between riskiness and successful firefighting). When participants then estimated the actual association in the information they were given, it was found that explaining multiple relationships resulted in more accurate perceptions of the available evidence. A follow-up study suggested that the effect emerges only when the alternative being considered is believable; when the alternative explanation was not viewed as plausible, no debiasing effect was found. Considering the alternative may then be a more appropriate intervention in contexts where considering the opposite is implausible or impractical (e.g., in cases where people only hold mild or neutral beliefs about a topic).

#### Linear Models

A second strategy for altering processing strategies is through “linear models” (Milkman et al., 2009). Here, more accurate judgments are achieved by asking people to either (a) restrict decision-making to only those criteria that are most relevant (i.e., reliably predict the outcome) or (b) use decision-formulas that apply different weights to various decision-making criteria. In one classic example of this latter approach, Dawes (1971) evaluated how graduate admissions were handled in his department. The admissions process required professors to synthesize across several criteria, which varied in scale (e.g., GPA versus GRE scores) and format (e.g., a transcript versus a recommendation letter). Traditionally, professors were asked to make a single, global judgment of each applicant on a 1 to 6 scale; applicants who received the highest average ratings across professors were then accepted. The author proposed a new approach: standardizing and summing each applicant’s undergraduate GPA, GRE scores, and rigor of undergraduate institution. This simplified method was a stronger predictor of graduate students’ first-year performance than the global evaluation typically used in the admissions process.

The linear models approach can extend to other domains. One example used the approach of restricting judgments to only the most relevant criteria (Samson & Thomas, 1987): while insurers have been known to use 12 or more variables when trying to arrive at rate estimates for drivers, using just four factors (driver age, area of residence, vehicle type, and history of filing claims) achieved comparable performance. Another example used real-world data concerning judges’ decisions of whether individual defendants should be released or held on bail before trial, with the risk being that released defendants may not appear for later court dates. In these cases, judges are free to use a variety of inputs when making their decision (severity of the crime, prior record, etc.). In an analysis of over 100,000 release decisions (Jung et al., 2020), a decision-making model using only two inputs (age and a prior history of missing court appearances) more accurately predicted future missed court appearances than judges’ actual decisions.

By simplifying the judgment criteria, the linear models approach reveals how decision-makers can arrive at less-biased behavior. However, it is crucial that applications of the linear models approach focus on using pre-established criteria that have been shown to reliably predict the outcome of interest (e.g., likelihood of future driving accidents for judgments of car insurance rates). That is, merely accentuating noisy or inaccurate criteria will not lead to superior decision-making; in one real-world example, variation in prison sentences given by US judges increased after laws were changed that allowed judges greater discretion and a wider range of inputs to consider when deciding on what criminal sentence to provide (Yang, 2014). Later in this review, we return to the issue of finding appropriate judgment criteria and note the unique challenges that may arise when looking to effectively use the linear models approach in contexts of intergroup discrimination.

### Change the Context

A final approach to debiasing judgment involves changing the judgment context by manipulating how relevant information is presented or how the decision-making process is constructed. Where the prior approaches sought to change some characteristic of the decision-maker (ability; motivation) or provide a superior strategy to navigate a judgment (change the decision-making process), this strategy seeks to change how decisions are presented and structured in hopes that certain formats will elicit more unbiased thinking or behavior.

#### Changing How Decisions Are Presented

Accentuating specific information is one way of changing the decision-making context. Consider the phenomenon of “reframing” (Tversky & Kahneman, 1983), which refers to manipulating how certain outcomes are highlighted in efforts to influence judgment. For instance, Sedlmeier and Gigerenzer (2001) improved participants’ logical reasoning by altering whether they presented information using probabilities (e.g., that 1% of women having a mammography show evidence of breast cancer) or frequencies (e.g., that 10 of every 1,000 women having a mammography show evidence of breast cancer). Participants trained using a frequency format showed stronger overall reasoning performance, less decay, and greater transference to new logic problems than participants trained using probabilities. The authors argue that frequency-based training may be superior because the format aligns with how people naturally think about the world; we are more likely to think about the *number* of people impacted by an event than the percentage of people impacted (see Van Roekel et al., 2022 for an application of this approach using field methods to increase handwashing behavior in a hospital). In related work, another study found that non-expert participants were able to make more accurate judgments about the benefits of a hypothetical drug when such information was presented with an “icon array” that visually represented the drug’s effectiveness in terms of frequencies (Garcia-Retamero et al., 2010). In these cases, certain types of information may be processed more clearly, and finding those preferred presentation formats can lead to more accurate judgment or more desired behavior.

Another method of altering how information is presented is to manipulate units. Research on the “MPG Illusion” (Larrick & Soll, 2008) reveals how consumers misunderstand fuel efficiency information when presented in miles per gallon (MPG). Specifically, people on average assume a linear effect of MPG on gas saved (e.g., that the difference in gas saved between a 20 MPG and 15 MPG vehicle is equal to the gas saved between a 50 MPG and 45 MPG vehicle), when in reality, the relationship is curvilinear: much more gas is saved when moving between lower levels of fuel efficiency (15-20 MPG) than higher levels (25-30 MPG). In one sample, only 1% of participants correctly identified how well various cars reduced their gas consumption when such information was presented in MPG (Larrick & Soll, 2008). However, a follow-up study found that a different unit, gallons per mile, lessened (but did not eliminate) this error. Similar framing effects have been found in other domains or contexts; for instance, coupons that highlighted a percentage of the original price (versus total money saved) were more attractive in a sample of Chinese consumers (Zhang & Han, 2012).

A final example concerns messages that accentuate gains versus losses. Prior work (Rothman & Salovey, 1997) has found that highlighting a potential loss is more effective at increasing behaviors associated with detection (e.g., getting a mammogram to find breast cancer) while highlighting a potential gain is more effective at increasing behaviors associated with prevention (e.g., wearing sunscreen to prevent skin cancer). In the study, participants read a pamphlet about gum disease that either advocated for a prevention behavior (rinsing with mouthwash) or a detection behavior (a disclosing rinse that makes plaque visible). Moreover, these behaviors were either framed as gains—people completing the behavior are “taking advantage” of a way to reduce plaque—or as losses—people not completing the behavior are “failing to take advantage” of a way to reduce plaque. Results found that intentions to use the product and requests for a sample depended on the match between behavior and frame: participants were more likely to use the product if receiving a prevention behavior/gain frame or a detection behavior/loss frame. Broadly, these data reveal another instance of how subtle changes to the presentation of information can influence judgments and shape behavior.

#### Changing How Decisions Are Structured

A more popular approach to altering the decision-making context is through work on choice architecture or “nudging,” which seeks to change behavior through more structural changes to the judgment process (Thaler & Sunstein, 2008). Though this approach shares many similarities with the work reviewed in the previous section, choice architecture tends to adopt more fundamental rather than surface-level alterations to the decision-making environment. A classic example concerns decisions about retirement savings, an area where people often fail to effectively save for the future. When given access to their full paycheck, employees may be susceptible to an anchoring bias (Tversky & Kahneman, 1974), using the first available information as an arbitrary benchmark. They then compare what is listed in their full paycheck to what is left after sending money to a retirement fund, an affective experience that then makes it unpleasant to save. One means of circumventing this negative feeling is to make savings decisions proactively, such as by pre-committing beforehand to automatically send a portion of one’s paycheck to a retirement account. A study testing this approach (Thaler & Benartzi, 2004) found that a prospective savings program more than tripled the amount of money that employees saved. By committing to saving before money was available, employees overcame an overreliance on present information.

Indeed, precommitment has proven to be a widely effective means for debiasing judgment, and the approach shares clear similarities with the linear models approach in that both methods look to reduce reliance on in-the-moment emotions or heuristics (e.g., Dawes, 1971). The existing evidence supporting precommitment has spanned multiple domains. In one study, students who precommitted to more evenly spaced deadlines for their class assignments outperformed those who were given more flexibility in when to turn in their work (Ariely & Wertenbroch, 2002). That is, those students who committed to cutting down on their potential flexibility for submitting assignments were less likely to suffer from the adverse effects of procrastination. A study in the health domain (Schwartz et al., 2014) focused on consumers already enrolled in a program that reduced the price of healthier groceries. Some program members were then given the option to forfeit these savings if they failed to increase the purchasing of such items over the next six months. Those who precommitted to risk losing their potential savings increased their purchases of healthier food by 3.5% compared to people who either refused the precommitment option or consumers in a control condition that were not enrolled in the savings program (Schwartz et al., 2014).

Another example of choice architecture is the use of judgment decoys, in which the mere presence of an inferior option is enough to sway decision-making. For instance, participants deciding between two products (e.g., Microwave A and Microwave B) that vary on relevant traits (weight, price, power), can be pushed toward preferring Microwave A with the addition of a third option that is comparable but clearly inferior to Microwave A, or pushed toward preferring Microwave B with the addition of an option that is comparable but clearly inferior to Microwave B (Ariely & Wallsten, 1995). The presence of a decoy facilitates an easy comparison between two of the available options, which simplifies decision-making for participants.

These studies reveal how judgment can be impacted by structural changes to the decision-making process or the number of options presented. However, perhaps the most common form of nudging is changing the judgment default. Defaults are a simple but highly effective strategy for altering judgment and behavior. Changing the default response has had large effects on behaviors like healthy eating (van Kleef et al., 2018), charitable giving (Goswami & Urminsky, 2016), savings (Cribb & Emmerson, 2016), and even organ donation (Johnson & Goldstein, 2003). Aside from capitalizing on people’s bias for inaction, defaults may work by suggesting a recommended course of action that decision-makers then use to infer the appropriate response (McKenzie et al., 2006).

“Nudging”—and choice architecture in general—is a diverse field that uses a range of approaches, and a full review of such work is beyond the scope of this paper. Moreover, recent analyses suggest that different choice architecture manipulations vary widely in effectiveness (Mertens et al., 2022; Szaszi et al., 2022), and may also be specific to contexts where people approve of being nudged (de Ridder et al., 2022). Researchers looking to adapt these approaches for intergroup contexts would be well-served to do a more in-depth analysis of their chosen strategy, but broadly manipulations of decision structure remain a valid approach for debiasing.

## Applying Debiasing Research to Intergroup Prejudice and Discrimination

The preceding section highlighted various strategies in the debiasing literature. Below, we review how these same approaches have been or could be applied to intergroup contexts. Specifically, we separate our review into three sections: what is unlikely to work, what has already been shown to work, and what could work. We define interventions that “work” as those that at minimum provide causal evidence of reducing discriminatory behavior or biased judgment in at least one context. However, as we note in our limitations section, the way we discuss what “works” for this review may not suffice for what “works” in real life: successful real-world interventions likely involve evidence from sources beyond mostly controlled, laboratory studies with non-expert, majority group participants and hypothetical or low-stakes outcomes. Nonetheless, we contend that by showcasing prior successful applications of various debiasing approaches to reducing discrimination, we can motivate researchers and practitioners to further apply this initial work to a wider range of populations, situations, and outcomes.

### What Is Unlikely to Work

For various reasons, certain debiasing strategies may be ineffective in reducing discrimination. Below, we review two common debiasing approaches that we believe will be unsuccessful in reducing intergroup biases in behavior: (a) abstract warnings or raising awareness and (b) financial incentives.

#### Raising Awareness

One naturally appealing strategy for debiasing judgment is to warn people about the possibility of bias, with the hope being that such warnings may translate into greater effort or the adoption of more successful decision-making strategies. Some evidence exists that this approach may be successfully adopted in studies on intergroup relations, specifically in changing intergroup perceptions or attitudes. For instance, Nasie et al. (2014) used an awareness-raising strategy to mitigate naïve realism in the context of the Israel–Palestinian conflict. In the experimental condition, Jewish and Palestinian Israeli students read a short text describing the phenomenon of naïve realism and how it functions in the context of conflicts. Relative to a control condition, results found that raising awareness of naïve realism increased right-wing Jewish and Palestinian Israeli participants’ openness to their adversary’s narrative (e.g., agreeing on such statements as “I think the [Palestinian/Jewish] attitudes regarding this issue are legitimate”). Thus, correcting for biases that maintain intergroup hostility may be one avenue for mitigating unintended discrimination (see Ehrke et al., 2020 for another example of raising awareness to change intergroup attitudes).

However, despite the allure of this approach, it has received only little empirical support in the literature on debiasing actual judgment. One example comes from prior work illustrating the effectiveness of the “consider the opposite” strategy (Lord et al., 1984), as participants in one experimental condition showed no reductions in motivated reasoning when they were asked to consider the evidence in front of them in an “objective and unbiased” manner. In another instance (Fischhoff, 1977), an overconfidence bias in trivia knowledge was unaffected when participants were told to “devote as much attention to this task as you can” or when reading an example of overconfidence and being told to “do everything you can to avoid this bias.” Raising awareness of bias and warning people to avoid it then seems like an unproductive means for debiasing judgment (see Larrick, 2004 for a similar conclusion on the limited appeal of awareness interventions).

In the context of discriminatory behavior, some evidence exists showing that generalized warnings are similarly ineffective. In Axt et al. (2018), participants completed a hypothetical admissions task known to produce discrimination favoring more physically attractive applicants. An intervention that informed participants of several potentially biasing factors—such as age, attractiveness, gender identity, or race—showed no changes in levels of discrimination relative to a control condition. In another example, participants were no less reliant on (irrelevant) facial characteristics like perceived trustworthiness when making hypothetical sentencing decisions after being told that perceptions of facial trustworthiness were unrelated to actual behavior and receiving a warning to not use such facial features in their decision-making (Jaeger et al., 2020). One explanation for the failure of general warning manipulations is that people may be largely motivated to avoid such biases but lack introspective insight into what must be done to achieve unbiased behavior; more effective awareness or education manipulations must instead provide decision-makers with information about the magnitude and direction of bias that needs to be counteracted (Wilson & Brekke, 1994).

#### Financial Incentives

Incentives have proven to have an inconsistent impact in the debiasing literature (for review, see Camerer & Hogarth, 1999). In general, incentives can influence behavior when the source of bias or poor performance is a lack of effort or motivation, and effects of financial incentives have emerged in areas like dieting and weight loss because these behaviors require high effort and motivation (Jeffery, 2012; Pasdar et al., 2021). However, many instances of intergroup discrimination may not come from a lack of motivation to regulate one’s potential biases (Bertrand et al., 2005).

Take, for instance, the concept of internal and external motivation to respond without prejudice (Plant & Devine, 1998), where internal motivation refers to the degree to which someone resists prejudiced thoughts or behaviors because of internal standards and values. Individual differences in the construct can be assessed using a self-report scale (e.g., “Being nonprejudiced is important to my self-concept”), and recent data suggests people, on average, carry an internal motivation to respond without prejudice in a sample of White undergraduates at an American university (Axt & Trawalter, 2017), 93% were above the scale midpoint when the internal motivation measure was about prejudices toward Black people and 85% were above the midpoint when the scale concerned prejudices toward people who are lower in physical attractiveness. Similarly, the 2021 data from Project Implicit’s racial attitudes task (Xu et al., 2014) found that, in a sample of more than 33,000 responses, 91% of participants were above the midpoint when reporting internal motivation to respond without prejudice toward Black people.

As mentioned earlier, a nontrivial portion of the population indeed has a desire to express prejudice (Forscher et al., 2015), and this intention likely carries over into intentional acts of discriminatory behavior (Campbell & Brauer, 2021). At the same time, the research reviewed in this section indicates that a large share of the population is motivated to regulate their potentially prejudiced reactions. In contexts where biased behavior emerges despite egalitarian motivations, discrimination likely arises for reasons beyond low motivation, such as an inability to translate the desire to be unprejudiced into concrete behavior or reliance on ineffective decision-making strategies. As a result, financial incentives are unlikely to be effective in changing discriminatory behavior. For instance, in a study using the same decision-making task concerning attractiveness biases, participants showed no reductions in discriminatory behavior when more accurate performance was rewarded with a financial incentive (here, a donation to a charity of the participant’s choosing; Axt et al., 2023).

### What Has Worked

The intergroup literature already has several examples of reducing prejudice or discrimination by drawing from broader work in debiasing. These studies provide “proof of concept” for the general approach that we are advocating for, and because judgment biases and intergroup biases may rely on shared psychological processes, it is possible for interventions to be successful in either domain. To be clear, only a small portion of the research reviewed below uses either real-world outcomes, naïve participants, or representative samples. These factors rightfully raise questions about the generalizability of the results. However, there is some available evidence to support a causal argument that each approach reduces discriminatory behavior—at least in certain contexts—and these prior studies are suggestive that such strategies could be successfully applied to real-world instances of intergroup discrimination.

We review several instances of prior work that has applied the debiasing strategies reviewed previously to an intergroup context, either through (a) increasing feelings of accountability, (b) requiring pre-commitment to decision criteria, (c) introducing judgment decoys, or (d) changing decision defaults.

#### Accountability

As reviewed earlier, feelings of accountability have successfully debiased judgment in a number of decision-making domains. Prior studies using undergraduate participants have found that increased accountability reduced reliance on primacy effects in interpersonal perception (Webster et al., 1996) or broadened the amount of information considered when evaluating hypothetical targets (Siegel-Jacobs & Yates, 1996), while related work found that accountability reduced reliance on heuristics in financial judgment among a sample of professional accountants (Fehrenbacher et al., 2020). Though certain conditions need to be met for accountability interventions to be effective, such as an ability to pay sufficient attention to one’s own decision processes (Kennedy, 1993), the many contexts in which accountability interventions have shown some impact make it a promising debiasing strategy.

Accountability has also been shown to be effective in intergroup contexts. For instance, in studies using the minimal group paradigm, increasing accountability by warning student participants that they would later need to justify their decisions reduced ingroup favoritism when allocating hypothetical rewards (Dobbs & Crano, 2001). Similarly, another accountability intervention lessened bias against Hispanic targets when undergraduates were tasked with judging the guilt of a hypothetical student accused of assault (Bodenhausen et al., 1994). A more recent study (Nadler et al., 2014) used an accountability intervention to improve evaluations of gay versus straight applicants in a mock hiring task.

Advocating for the use of accountability in addressing discrimination presents a conceptual challenge because accountability is often discussed as a means of changing motivation (Lerner & Tetlock, 1999), and we have previously argued that since motivations to regulate prejudice or discrimination are already high, interventions targeting motivation are then unlikely to be effective in an intergroup context. We believe this inconsistency can be addressed by differentiating between one common effect of motivation-related interventions—using the same strategy but now with greater effort (John et al., 2011)—with a separate effect of motivation-related interventions—the adoption of novel, more complex decision-making strategies.

In the context of intergroup discrimination, motivation-related interventions that are primarily associated with increasing effort (e.g., financial incentives; Axt et al., 2023) will be mostly ineffective, since both student and online samples (at least in North American contexts) typically self-report report high levels of internal motivation to control prejudiced behaviors (e.g., Hester et al., 2023; Lacosse & Plant, 2020). However, accountability interventions may derive their effectiveness in intergroup contexts from this second mechanism: the use of more nuanced and thorough processing strategies (e.g., Webster et al., 1996), potentially due to greater feelings of self-criticism (Lerner & Tetlock, 1999). While we believe this interpretation is consistent with the available evidence, additional research is needed that can more concretely identify why accountability interventions have consistently reduced discrimination where other motivation-related interventions, such as raising awareness or providing financial incentives, have failed.

#### Precommitment

Making decisions in advance, or committing to using specific criteria beforehand, has proven to reduce biases (at least in the short term) for real-world decisions related to savings (e.g., Thaler & Benartzi, 2004), health-related choices (see Reisgies et al., 2023 for meta-analysis estimates) as well as in hypothetical outcomes like potential policy support (Rogers & Bazerman, 2008). Precommitment strategies have also been shown to impact judgment or behavior in non-Western samples (e.g., Schilbach, 2019; Witvorapong & Watanapongvanich, 2020).

Precommitment has also been effective in the context of discrimination. In one well-known study (Uhlmann & Cohen, 2005), undergraduate participants were given the task of hiring the next police chief and were presented with one male and one female applicant. Between conditions, the strengths of these applicants were crossed; in one condition the male applicant was described as “streetsmart” and the female applicant as “well-educated,” with these labels switched in the other condition. When applicant gender and qualifications were presented simultaneously, participants on average favored the male applicant, regardless of qualifications. However, gender-based discrimination was reduced in a condition where participants had to precommit to their evaluation criteria before seeing the applicants (i.e., deciding whether being streetsmart or well-educated was more important for the position).

More recently, precommitting to using more objective criteria when choosing a partner in a hypothetical trivia task (e.g., a partner’s prior performance in trivia) lessened biases in selection based on gender, country of citizenship, or weight (L. W. Chang & Cikara, 2022). Similarly, in a contest design that tested 30 different interventions for reducing attractiveness-based discrimination in a mock admissions task among online, volunteer participants (Roy et al., 2024), the most successful interventions were those that prompted participants to commit to using specific evaluation criteria beforehand, either by using criteria provided by the researchers or by generating one’s own criteria.

Precommitment interventions may work in the intergroup context by heightening the need for consistency between one’s values and behaviors (e.g., Festinger, 1957); decision-makers will want to stay consistent with their precommited criteria, and this desire can override the biasing effect of demographic information like race or gender.

Despite the usefulness of the strategy, precommitment is not popular in the discrimination-reduction literature, as our review found very few studies in the last 5 years that used this approach in an intergroup context. One reason for this hesitancy could be that while precommitment potentially reduces biased judgment by increasing the accuracy of decision-making, many criteria could exacerbate existing systemic discrimination. For instance, if a bank officer precommits to payment history as a criterion when deciding who to offer a loan, this criterion, while seemingly objective, could be biased against Black people who are statistically less likely to have high credit scores due to unjust policy practices in the past (e.g., redlining). Precommitment alone may then be insufficient to address discrimination. Nonetheless, researchers interested in reducing discrimination should continue to consider precommitment as a potential avenue, especially if they are confident that evaluators could precommit to relying on unbiased, decision-relevant criteria.

#### Judgment Defaults and Decoys

Choice architecture is a broad approach to debiasing that involves shifting behavior by altering how a decision is structured. One common intervention in this area involves changing choice defaults (Goswami & Urminsky, 2016); indeed, a recent meta-analysis found that choice default manipulations produced an overall effect of *d* = .68 on participant judgments, with results not being moderated by whether the study took place in the United States (though results were weaker when dealing with judgments related to environmental behavior; Jachimowicz et al., 2019). A related approach concerns presenting decoy options (Wedell & Pettibone, 1996), which can shift preferences to certain options and has shown effects across a variety of participant populations (e.g., Park & Kim, 2005; Pittarello et al., 2020; Wu et al., 2020).

Recent work has shown that each of these methods can also be useful for combating discrimination, particularly gender-based discrimination. For instance, L. W. Chang and Cikara (2018) found that introducing a decoy option in the judgment process aided the evaluation of counter-stereotypical applicants among online participants completing a hypothetical hiring task (e.g., a woman rated lower in warmth; see Keck & Tang, 2020 for another application of decoy effects in gender-based discrimination). Another series of studies leveraged the power of defaults and was able to strongly reduce gender-based disparities in decisions on whether to compete; across multiple contexts, in both lab and field studies, women were just as likely as men to enter competitions when the choice to compete was made the default (He et al., 2021).

Other approaches in the choice architecture literature have also been successfully applied to intergroup discrimination. For instance, in building off of prior work on the effects of joint vs. separate evaluation (Hsee et al., 1999), discrimination against women in a hypothetical academic exercise was reduced when women were evaluated simultaneously (i.e., on the same screen) alongside men compared to when men and women were evaluated one at a time (Bohnet et al., 2016). Related work has reduced discrimination by introducing other structural changes to the decision-making process. In one study, both adult and undergraduate participants were more likely to ultimately nominate a woman for a hypothetical technology position when required to first generate a shortlist of six applicants compared to a condition that only shortlisted three applicants (Lucas et al., 2021). Elsewhere, gender-based discrimination was reduced in a mock hiring task when online participants were asked to fill several positions versus a single position (E. H. Chang et al., 2020).

These studies represent some of the most exciting recent developments in work on reducing discrimination, and we believe that many future avenues exist for applying work in choice architecture to the issue of intergroup bias in behavior. For one, these studies have focused largely on gender, and similar gains may be found when applying the same strategies to other prominent areas of discrimination, such as based on race or age. In addition, other strategies from the nudging literature may be adopted for intergroup contexts, such as introducing planned interruptions when judging who to admit during college admissions or “fresh-start” framing to sustain motivation to act in a nonprejudicial way (Duckworth et al., 2018). While there is a clear record at this point for choice architecture strategies as being effective routes to reducing discrimination, we are optimistic that there are still many unexplored insights from the general nudging literature toward the issue of discrimination.

### What Could Work

Our final section highlights areas of debiasing research that we believe could be productively applied to reducing discrimination. In many cases, such strategies have support from existing research in the prejudice and discrimination literature through the use of related methods or outcomes. These interventions focus on changing an individual’s ability or motivation (e.g., focused training), changing the processing strategy (e.g., considering the opposite), or changing the decision context (e.g., making decisions between individuals vs. groups).

#### Training

As reviewed earlier, training has an inconsistent record in debiasing judgment (Schwitzgebel & Cushman, 2012; Wagenaar & Keren, 1986). These mixed results are also likely to be true of training meant to target discriminatory behavior. Indeed, field studies have shown that broader bias training programs have often failed to impact subsequent behavior (E. H. Chang et al., 2019; Kalev et al., 2006). Given the equivocal findings, a better question seems to be not *whether* training can debias judgment but *what type* of training does so.

Drawing again from Fischhoff’s (1982) tenets for effective training, we believe that training programs are likely to reduce discriminatory behavior when they (a) allow for practice, (b) have clear outcomes, and (c) provide feedback or reward. Unfortunately, many instances of intergroup discrimination are unlikely to meet these criteria; for example, while hiring managers may have sufficient practice in evaluating applicants, they often lack clear evidence of a successful hire, as it can be difficult to attribute an employee’s failure to personal factors (e.g., poor work ethic) or contextual factors (e.g., an unsupportive work environment). Managers also may not have the opportunity for sufficient feedback, as it is impossible to know how unselected applicants would have performed if hired.

Though achieving these conditions may be difficult, training that do so is likely to be beneficial. For instance, in the research contest comparing interventions for reducing attractiveness-based discrimination in a hypothetical decision-making task, the single most effective intervention was one that asked participants to practice evaluating novel applications and provided immediate feedback as to whether they had arrived at the correct judgment (i.e., had properly evaluated the applicant’s qualifications; Roy et al., 2024). In another example, reliance on facial stereotypes (i.e., facial features associated with trustworthiness) was lessened in an economic trust game (where participants’ performance could impact actual study payment) when preceded by training that showed targets with faces rated as less trustworthy engaging in clearly trustworthy behavior (Chua & Freeman, 2021). In all, these data suggest that training can be effective so long as they are specific and geared toward how to best navigate the judgment at hand, while broader training that overlook more concrete information and feedback is less likely to impact intergroup biases in behavior.

That said, there is still some potential to reduce discrimination through more generalized training, specifically ones that highlight structural similarities across a number of biases. In particular, a greater conceptual understanding of how various biases share certain properties may promote transference effects as well as greater efforts to avoid biased judgment. One example of this strategy comes from research on analogical reasoning (Thompson et al., 2000), which trains people to understand the common principles that give rise to various behaviors. Training in analogical reasoning has been shown to debias judgment in several contexts. For example, Hungarian undergraduate participants (Aczel et al., 2015) who engaged in a group discussion over the similarities that exist in numerous judgment biases (e.g., base rate neglect and sunk cost fallacy) showed superior performance in novel measures of judgment bias that were not discussed (e.g., framing effects and overconfidence biases), and this manipulation outperformed an awareness manipulation that merely reviewed the biases (see Idson et al., 2004 for a laboratory study among American participants showing other transference effects of training on biased judgment). Analogical reasoning may then provide participants with a broader ability to identify the situational factors that create bias and, as a result, be less impacted by such factors.

Similar analogical reasoning strategies could effectively reduce discrimination, though these efforts likely require going beyond general warnings about bias (Axt et al., 2018). For instance, it is possible that an analogical reasoning intervention could focus on the structure and consequences of bias against people with disabilities in a hiring context and then expand that discussion to other contexts, such as in dating, health care, and housing. Alternatively, training that focused on the shared properties of many forms of intergroup discrimination (e.g., based on race, age, or religion) could allow participants to rely less on other demographic factors (e.g., gender or sexual orientation) in novel contexts.

#### Consider the Opposite

In our view, there are no interventions in the prejudice or discrimination-reduction literature that could be considered close uses of the “consider the opposite” strategy (Slovic & Fischhoff, 1977). However, the abundance of laboratory evidence on student samples shows that this approach has worked to lessen overconfidence biases (Koriat et al., 1989), confirmation biases (Lord et al., 1984), and hindsight biases (Arkes et al., 1988). When appreciating this work alongside other studies that have shown positive results of considering the opposite in populations like employee managers (Nagtegaal et al., 2020) or police officers (Fahsing et al., 2023), there is reason for optimism that the approach could be effective for intergroup biases as well.

As one example, discrimination stemming from social factors like race or gender could be lessened in a hiring context if, for every judgment, people needed to play the “devil’s advocate” before coming to a final judgment. In this case, if a hiring manager was leaning toward rejecting an Indigenous applicant and hiring a White applicant, it may be helpful for the manager to then articulate why the opposite decision may be preferable by listing reasons why the Indigenous applicant should be hired and the White applicant rejected. These exercises could lead to a more consistent decision-making process and allow evaluators to attend to strengths or weaknesses that may have been initially overlooked. In all, considering the opposite could then reduce reliance on initial preferences that may be driven by irrelevant factors, like ingroup status.

Though the exact strategy of “consider the opposite” has not been used in the prejudice or discrimination literature, the success of related approaches bolsters our belief that “consider the opposite” will be a potent intervention. One recent intervention strategy in prejudice research comes from “paradoxical thinking,” which involves presenting individuals with statements that, while broadly consistent with their held beliefs, are consistent in “an amplified, exaggerated, or even absurd manner” (Hameiri et al., 2019). Being confronted with logically consistent but exaggerated versions of one’s thinking can prompt a reexamination of key assumptions in reasoning, often leading to changes in beliefs and behavior. This perspective has been shown to be successful in an intergroup context. In one example (Hameiri et al., 2014), Israeli participants who watched a video espousing exaggerated pro-Israel statements (e.g., “we need the [Palestinian] conflict to have the strongest army in the world”) showed greater willingness to compromise with Palestinians, and these effects lasted up to a year. Subsequent studies using this same manipulation found that the paradigm also led to more charitable attributions toward Palestinian people and created a greater willingness to consider alternative information about the Israeli-Palestinian conflict (Hameiri et al., 2018). More recently, Knab and Steffens (2022) showed effects of a paradoxical thinking intervention on reducing intergroup hostility using a separate context (Germany) and target group (refugees).

A related approach to reducing intergroup prejudice is through feelings of hypocrisy (Bruneau et al., 2018). In studies using this strategy, participants first advocate for a position but are then shown how their own beliefs fail to adhere to these standards. For instance, participants may broadly endorse the notion that it is unfair to judge an entire group of people because of the behavior of a small subset of that group but do just this when forming negative impressions of all Muslims based on the actions of a violent subgroup. Having one’s hypocrisy highlighted likely induces feelings of dissonance, which people resolve by altering their intergroup attitudes. Indeed, participants who underwent a hypocrisy intervention showed reduced blame toward Muslims both immediately and 1 year after the study session (Bruneau et al., 2020).

Both paradoxical thinking and hypocrisy induction share broad similarities with “consider the opposite” in that they prompt participants to engage with beliefs counter to their initial reactions. However, we believe that considering the opposite could be more potent in the context of discrimination because hypocrisy induction and paradoxical thinking operate by changing attitudes (i.e., by prompting the reexamination of beliefs in hypocrisy induction or by promoting cognitive dissonance in paradoxical thinking), consider the opposite targets behavior change more directly by allowing the opposing outcome to be more easily accessible (e.g., having to consider whether a preferred applicant should actually be rejected before making a final judgment). More broadly, given the success of the “consider the opposite” interventions in many domains of judgment decision-making, we believe that further adaptions of prior uses of the method (e.g., Koriat et al., 1980; Mussweiler & Strack, 2000) could become effective manipulations for reducing discrimination in a variety of contexts.

#### Consider the Alternative

At their core, “consider the alternative” interventions work by encouraging people to simulate different outcomes (Hirt & Markman, 1995). Similar strategies have been effective in intergroup contexts, specifically for prejudice reduction. For example, interventions that ask participants to imagine experiences of contact with members of another group have created more positive intergroup attitudes and lessened intergroup anxiety (Crisp & Turner, 2009; Prati et al., 2015; Turner et al., 2007). Similarly, counterfactual thinking—where people generate alternative outcomes for past events (Roese, 1997)—has been applied to intergroup prejudice (Winter et al., 2023). In one study using a counterfactual thinking manipulation (Miller et al., 2013), a sample of American undergraduate participants were asked to imagine a scenario in which they had a pleasant interaction with a gay man and then later learned that this man was actively discriminated against in school. Those asked to think about how this discrimination could have been avoided showed lower levels of self-reported prejudice toward gay people. The success of “considering the alternative” in judgment and decision-making contexts, as well as the effectiveness of related approaches like imagined contact or counterfactual thinking, indicates that the strategy may be useful in other intergroup contexts; for example, there may be transgender applicants for an audition for a role in a television series, and casting directors, when deciding whether or not to hire them, can consider what they would do if those applicants were cisgender.

However, while there are some reasons to be hopeful about strategies like considering the opposite or considering the alternative being effectively applied to discrimination, it is worth noting that these interventions take a more “mentalizing” approach, in that they seek to change how people think about a decision or group. There is emerging evidence in the social psychology literature that targeting such processes may be an ineffective means for creating sustained change in intergroup processes, specifically in regard to stereotypes (Lai et al., 2016; Paluck et al., 2021) as well as outcomes like awareness of bias (Devine & Ash, 2022). This work raises doubts about whether other interventions that look to target participant thinking styles will show lasting effects on changing discrimination, and more fruitful efforts may come from strategies that avoid this issue and instead focus efforts on other approaches, like manipulating how decisions are framed. However, our focus on unintended discriminatory behavior (rather than intergroup prejudices or beliefs) may be a more productive outcome given the previously reviewed studies showing that intergroup behaviors can often change without corresponding changes in prejudices or stereotypes (e.g., Mousa, 2020; Scacco & Warren, 2018).

#### Linear or Algorithmic Decision-Making

Another debiasing strategy that could be applied to discrimination reduction is linear models or algorithmic decision-making, where complicated multi-attribute decisions are reduced into simpler judgments. We believe there is some promise for this approach in addressing intergroup discrimination but at the same time caution that these strategies may prove ineffective or even detrimental if not applied thoughtfully.

Our hesitancy in recommending these approaches echoes our earlier criticism of relying on precommitment as a discrimination-reduction strategy, as each requires the use of pre-established decision-making criteria. However, if the decision-making inputs guiding judgment are themselves flawed, then using that information will only replicate or exacerbate existing intergroup disparities. Several recent studies have highlighted this issue in terms of using computer algorithms to make decisions about individual treatment. One real-world example of this issue used a hospital that relied on a computer algorithm to decide which patients deserved to be included in a new drug trial (Obermeyer et al., 2019). Though the algorithm did not directly include patient race when arriving at a recommendation, the output still reproduced racial disparities such that, for a Black and White patient experiencing equal symptom severity, the algorithm was more likely to suggest the White patient be entered into the drug trial. Here, the issue was that the algorithm decided to use health *costs* as a proxy for general health, and issues unrelated to actual health can lead to White patients spending more money on their health care than Black patients (e.g., available income, flexibility in work schedules to attend appointments). When a biased measure is treated as an objective indicator, disparities will persist. These concerns are only amplified by the fact that people are less bothered by discrimination created by algorithms than they are by discrimination created by human beings (Bigman et al., 2022).

We believe the same concerns apply to using linear decision-making to reduce discrimination. Consider the previously discussed work on insurance adjusters (Samson & Thomas, 1987), which found that equally accurate performance could be achieved by simplifying judgments from using twelve to four variables when deciding on what factors to use in generating car insurance estimates. Two of the four retained variables were area of residence and whether the applicant had a history of filing claims. In an intergroup context, it is plausible that these inputs are biased. For example, companies may provide higher insurance rates to racial minorities because they are more likely to live in low-income neighborhoods. Or, low-socioeconomic status (SES) policyholders may be given higher rates because they file more claims than high-SES policyholders, who can pay for expenses out of pocket. Similar processes could certainly occur in other consequential domains. Admissions officers who remove demographic information and instead focus on supposedly objective indicators of quality may then fail to consider how such outcomes are impacted by possible intergroup disparities; for instance, ignoring SES could be problematic when weighing extracurricular activities, since low-SES students will be disproportionately more likely to work afterschool jobs.

In one sense, following algorithmic judgments or linear decision-rules may lead to less-biased judgment in the strict sense that decision-makers will be less impacted by the motivated, incomplete, or flawed processing of available information. But in many contexts that hope to reduce biased judgment through greater reliance on “objective” measures, doing so will only create, uphold, or increase discrimination, since the supposedly objective metrics are picking up on intergroup disparities that are the product of more structural factors (e.g., pre-existing wealth gaps between racial groups). In these cases, individuals will have to decide whether introducing some counteractive measures into the evaluation process is consistent with personal and organizational values; for instance, some insurance companies may be willing to remove an applicant’s history of filing claims from the evaluation process (thereby technically introducing noise into judgment) if doing so reduces racial disparities in insurance rates.

Despite our general hesitancy, there is a reason for cautious optimism in the use of algorithmic or linear decision-making for addressing discrimination. One example comes from a recent study on college admissions (Bastedo et al., 2022), which investigated the benefit of an “environmental context dashboard” for admissions officers viewing applicants. Most admissions programs rely on holistic admissions, where officers synthesize a range of relevant information spread over several pages to arrive at a global evaluation. The dashboard intervention, however, presented the most relevant information more succinctly. Specifically, the dashboard displayed how each student’s standardized test scores compared to other students from the same high school, and also presented demographic data from that student’s neighborhood (e.g., median income, percentage of students who have access to stable housing). This approach may be an effective means of neutralizing the previously discussed criticisms of a linear models approach, as it contextualizes these “objective” measures of student quality (e.g., an SAT score that is average for the entire applicant pool may be exemplary when considering other SAT scores from the applicant’s school district). Indeed, in a randomized trial (Bastedo et al., 2022), the dashboard made admissions officers more likely to recommend applicants from lower socioeconomic backgrounds. Similarly, an archival analysis of over 3.5 million applications across 43 schools found that presenting student qualifications with more contextual information was associated with a 5% increase in the probability that an applicant from a lower socioeconomic background would receive an offer of admission (Mabel et al., 2022).

Simplifying judgment by lessening the information considered during decision-making may effectively reduce discrimination, but caution is required when deciding which information to retain. In particular, researchers must avoid including variables that will only reproduce existing intergroup disparities.

#### Altering Presentation Format

A final debiasing strategy that can address discrimination comes from manipulating how information is presented, such as by altering how the consequences of discrimination are framed or changing what information is accentuated during evaluation. This approach builds from prior debiasing work, revealing how decisions can be sensitive to even small changes in frames or judgment units (Larrick & Soll, 2008; Sedlmeier & Gigerenzer, 2001).

There is some preliminary evidence to suggest that interventions altering framing are a good fit for the topic of discrimination. For instance, prior studies have shown differing patterns of behavior when people are asked to evaluate *individuals* versus *policies* that impact larger groups. In one example (Munguia Gomez & Levine, 2022), college admissions officers were asked to choose which of two applicants should be prioritized in a hypothetical admissions task: one that had higher test scores and came from a more privileged background (indicated by household income) versus one with lower test scores that came from a less privileged background. The officers were more likely to admit the privileged student when only selecting between the two applicants but were more likely to select the less privileged student when the same students were framed as representing the *types* of applicants that should be prioritized in the broader admissions process. In other words, the more privileged student was prioritized when selecting an individual applicant, but the less privileged student was prioritized when deciding what larger groups of students should be favored in admissions policies. Follow-up studies by Munguia Gomez and Levine (2022) found similar framing effects among working professionals when the context was gender biases in the tech industry. These effects were driven by shifts in thinking about fairness; specifically, considering policies versus individuals led to more “macrojustice” concerns about diversity. Applying this same framework to other forms of discrimination may likely be effective, as thinking about larger evaluation systems may reduce biases in individual judgments (Simonsohn & Gino, 2013). For example, when deciding among who to admit into a medical trial, doctors could be encouraged to think about the groups that may benefit most from treatment (e.g., intersectional groups like Black women, who have higher rates of cardiovascular disease than White women; Benjamin et al., 2018) versus thinking about which individual patients should be admitted.

This recent work follows studies in the intergroup literature using related outcome measures that fall short of directly investigating discriminatory behavior. For example, both lab and field samples revealed that support for policies seeking to reduce inequality was greater when the issue was framed as reducing disadvantages compared to reducing advantages (Dietze & Craig, 2021), an effect that was particularly likely to emerge when policies accentuated benefits to minority groups (see Brown et al., 2023 for additional field data on the effects of framing on race-related beliefs and motivations). There are many possible future directions for interventions that extend this line of thinking; for example, leasing officers’ may show less racial bias in judgments when decisions are framed as whether the applicant should be *denied* a housing opportunity versus whether the applicant should be *granted* a lease.

At the same time, a recent study (Bruckmüller & Braun, 2020) highlights a potential downside to this approach. Specifically, two samples of online participants found that framing gender inequality in the workplace as an issue of women being underrepresented (compared to men being overrepresented) led to explanations of inequality that focused more on women’s actions than on men’s, and created preferences for corrective policies that were targeted to women rather than pursuing more structural changes. Maximizing the benefits of such framing manipulations while minimizing any adverse effects is a clear goal for future work in this area.

More broadly, attempts to address discrimination may be aided by closer attention to framing effects. For instance, lab studies applying construal-level theory have found that invoking more abstract mindsets can weaken prejudice (Luguri et al., 2012; Yogeeswaran & Dasgupta, 2014), and creating these abstract mindsets through the framing of issues or behaviors could show similar effects in reducing discrimination. Other work has found evidence of framing effects in terms of whether choices are presented as either acceptances or rejections (Perfecto et al., 2017). Given the findings discussed above, we believe these subtler approaches to judgment formatting are underexplored in studies looking into discriminatory behavior. Future work may be able to apply these insights directly, such as by examining whether discrimination can be weakened when moving from a frame of whether an applicant should be accepted to whether an applicant should be rejected.

We acknowledge that some of these approaches may be challenging to implement in many contexts, but the importance of addressing an outcome like discrimination may merit and reward some innovative thinking. For instance, reframing evaluation decisions to be about why applicants should be rejected (or fired, or denied) versus why applicants should be accepted (or hired, or promoted) may be difficult, but decision-makers could be persuaded to pursue them if shown prior evidence that such changes could limit biased judgment. Even more drastic approaches, like intentionally adding decoy candidates to an applicant pool, could be welcomed if evaluators agree that such changes will help achieve the individual and organizational goals of less socially biased judgment.

## Future Directions

The present work explored how research in debiasing within relatively less social contexts (e.g., judgment heuristics) could be applied to studies looking to reduce intergroup discrimination. We reviewed fundamental strategies in the debiasing literature and then highlighted instances where such strategies have already been applied to the question of discrimination, before speculating on how underused strategies may or may not be successful in future research on the topic. However, while we believe our outcome of unintended discrimination in judgment is a key contributor to creating or maintaining intergroup disparities, there are other important forms of discrimination that are outside the scope of this work (e.g., lower feelings of inclusion for members of marginalized groups; Shore et al., 2011).

In our final section, we discuss current challenges in research on both debiasing and discrimination that we believe hinder communication between the two literatures. Specifically, we review several areas where the two fields lack common organizing frameworks and assumptions and how future efforts could begin to resolve these discrepancies. We also briefly suggest other research programs that could aid discrimination-reduction efforts.

First, debiasing and discrimination-reduction literature often rely on different levels of analysis when discussing some of the root causes of biased judgment or behavior. In particular, research in discrimination has begun to better appreciate how the phenomenon is shaped by systemic factors. For example, the previously discussed study on algorithmic racial bias in a medical context (Obermeyer et al., 2019) is reflective of how race-neutral policies can sustain intergroup disparities when they rely on inputs that capture structural forms of discrimination (e.g., differences in income between racial groups that allow one group access to better medical treatment). A similar process could emerge when using human evaluators; for instance, real-estate agents may unintentionally discriminate against people with disabilities when asking for proof of employment, as people with disabilities are statistically more likely to be unemployed and rely on government funding due to medical reasons.

The role of such systemic factors in contributing to discrimination is becoming more prominent in work on intergroup processes, and recent studies have even sought to formally tease apart what aspects of discrimination are driven by individuals versus systems (e.g., Barron et al., 2022; Bohren et al., 2022). We believe a similar approach would benefit the debiasing literature, which currently over-emphasizes the degree to which errors in judgment and decision-making are caused by irrational or imperfect thinking at the individual level, and fails to consider how these biases may be shaped by more environmental, systemic forces. Greater alignment between the two literatures may then emerge from less focus on fixing an individual’s faulty cognitions and more focus on *why* people have such faulty cognitions, a process that can have both individual and structural roots.

A second means of better integrating the debiasing and discrimination-reduction literature is through less use of “System 1” vs. “System 2” terminology in debiasing research. Classically, System 1 refers to mental processes that are comparatively automatic and require little cognitive resources, whereas System 2 refers to mental processes that are more deliberate, controlled, and dependent on cognitive resources (Stanovich & West, 2000). Reliance on this distinction between System 1 and System 2 remains popular in debiasing research (e.g., Boissin et al., 2022; Lawson et al., 2020), and a recent review of the debiasing literature also highlighted the risk of too much investment in dual-process models of judgment (Korteling et al., 2021).

Though intergroup discrimination research seldom uses the terms “System 1” and “System 2,” we can draw a clear parallel between this framework and the equally well-known distinction between “implicit” versus “explicit” cognition (De Houwer et al., 2009), with implicit cognition being more automatic and less aligned with conscious intentions compared to explicit cognition. Similar to research on “System 1” vs. “System 2,” classic studies in intergroup relations (e.g., Dovidio et al., 2002) have sought to situate implicit (i.e., indirect) measures as more predictive of relatively automatic components of behavior (e.g., nonverbal behavior) and explicit (i.e., self-reported) measures as more predictive of relatively controlled behaviors (e.g., self-reported intentions of friendliness). However, this dissociation has failed to receive consistent support in subsequent years. For instance, one meta-analysis looking at how well intergroup behavior was predicted by both self-report and a popular indirect measure, the Implicit Association Test (IAT), found that the IAT was just as predictive of behaviors rated as more versus less controllable (Kurdi et al., 2019), results that contradict the assumption that indirect measures more closely correspond to less deliberate behaviors.

Indeed, the current literature lacks a replicable behavioral measure that has been shown to be consistently related to implicit but *not* explicit measures of intergroup attitudes or prejudices (Axt et al., 2020). In light of this more recent evidence, researchers are increasingly deemphasizing the question of whether discriminatory behavior is driven by “implicit” or “explicit” biases. Given these changes in the discrimination literature, debiasing research may also want to focus less on targeting System 1 vs. System 2 thinking and instead achieve better alignment with the discrimination literature by emphasizing other potential mechanisms (e.g., whether interventions work by increasing effort or forcing a reexamination of existing beliefs). Doing so could reveal new intervention methods that discrimination research can later adapt.

An additional consideration for facilitating communication between the debiasing and discrimination-reduction literature is more attention to taxonomy. Ironically, the debiasing and discrimination literature appear to suffer from opposing shortcomings in their approaches to taxonomy. Specifically, while judgment and decision-making researchers have succeeded in identifying many unique biases, they have failed to reach a consensus on how to best label or classify such biases (though see Arkes, 1991 for one such attempt). As a result, the plethora of judgment biases in the literature risks a “jangle fallacy” (Kelley, 1927), where researchers treat conceptually similar processes as different because they have distinct labels. For example, researchers have sought to differentiate the phenomena of a “bias blind spot” (Pronin et al., 2002)—the tendency to see oneself as less biased than others—from that of “naïve cynicism” (Kruger & Gilovich, 1999), where people anticipate more egocentric biases in others than themselves. Though these processes could differ in subtle and potentially important ways, researchers should not treat them as separate by default, as the two biases could be manifestations of a single psychological force. Though there have been some ongoing attempts to create more unified taxonomies of biased judgment (e.g., Oeberst & Imhoff, 2023; Shah & Oppenheimer, 2008; Broder & Newell, 2008), these models have not been widely adopted. More broadly, research into judgment biases and debiasing could benefit from less attention on identifying unique biases and more attention on the structural similarities between biases; doing so would open avenues to tackle multiple biases simultaneously.

Conversely, the discrimination-reduction literature suffers from the inverse problem of too *little* recognition of the many different forms of discriminatory behavior. While there have been some attempts to differentiate between various types of discrimination, these efforts have relied on more basic distinctions, such as between intentional and unintentional discrimination (e.g., Bertrand et al., 2005). In this sense, research in discrimination can fall prey to the “jingle fallacy” (Kelley, 1927), where conceptually different processes are viewed as similar because they share a common label. For instance, researchers have labeled a wide range of behaviors as “discrimination,” such as selecting applicants for a hypothetical academic program (Axt et al., 2019), allocating financial support to various student groups (Pacilli et al., 2013), or deciding how close to sit to another person (Vartanian et al., 2016). While each of these behaviors fits into a broad definition of discrimination, they likely rely on different psychological mechanisms and considerations. Thus, a more thorough taxonomy of discriminatory behavior will help researchers better identify the contexts in which certain interventions are likely to generalize. For example, researchers could distinguish between discriminatory behavior driven more by affective processes like disgust from those driven more by motivational processes like confirmation bias, and reveal that some interventions, such as invoking accountability, are more impactful on motivational than affective forms of discrimination.

A related issue raised by a reviewer is that future research could benefit from exploring whether specific interventions are more appropriate for certain contexts. For instance, an intervention that targets an individual’s decision-making process (such as considering the opposite) might be most suitable for reducing unintended discrimination where decisions are allowed longer timeframes and more attentional resources can be allocated (e.g., deciding which employees may need to be laid off at a small company). On the other hand, interventions that target the decision structure (such as by pre-committing to judgment criteria) may be more appropriate for larger-scale decisions that require quicker decision-making and can incur more noise (e.g., when deciding among thousands of potential applicants for admission to a university). It will also be important to take into account that some interventions are more scalable than others; for instance, targeted training can be implemented online and efficiently given to a large audience relative to more technically sophisticated interventions like judgment decoys. However, more effortful interventions may be nonetheless useful for highly consequential decisions (e.g., who to hire for an executive leadership position).

Finally, while we have focused on the debiasing literature in this review, discrimination research can also adapt findings or ideas from other areas of the humanities and social sciences, as an interdisciplinary approach may reveal novel insights on lessening discriminatory behavior. One potential avenue is through recent work in philosophy on the difference between structural versus individual interventions. For example, while researchers in psychology are increasingly devoting more focus to the structural factors that create and maintain intergroup disparities, this approach may downplay the importance of changing individual minds as necessary for creating such structural changes (Madva, 2016). Giving more attention to work on this issue in philosophy may help researchers more fully appreciate what aspects of discrimination are being targeted by their chosen interventions.

Similar insights may emerge by attending to work in related fields, such as economics or operations management. These areas provide evidence—often in real-world contexts—concerning the success of other interventions in reducing intergroup discrimination, like the impact of prolonged intergroup contact (Rao, 2019), injunctive norms (Fang et al., 2019), or reputational information (Cui et al., 2020). Thus, greater awareness of efforts in other literatures can add nuance to our understanding of existing interventions in the psychological literature, or even introduce novel strategies for alleviating discrimination. Researchers in psychology may be particularly well-suited to build off research in other fields by exploring the mechanisms through which such interventions derive their effectiveness.

## Limitations Arising From Constrained Generality, Author Positionality, and Citation Practices

As mentioned previously, a large majority of the data reviewed in this article emerges from mostly White, Western, and younger samples. The relatively limited nature of these samples directly weakens any claims about whether certain judgment biases exist across other populations, and whether interventions targeting biased behavior will have similar effects when applied toward various groups (e.g., discrimination based on sexual orientation or disability status). Research looking to apply debiasing research to questions of discrimination will then benefit greatly from future work that expands both the diversity of participants and the diversity of study targets. In addition, even the literature that is cited fails to fully appreciate other important aspects of participant identities; for instance, none of the reviewed research considers intersectional identities and explores the unique effects that emerge from combinations of social identities. We recognize that intersectional identities are important. Much of the research in this review only tackles reducing discrimination across one identity (e.g., race or gender), but it is possible that reducing discrimination across multiple stigmatized identities requires different intervention mechanisms or strategies.

Furthermore, both authors have sociodemographic characteristics that were likely to impact our conceptualization of constructs like intergroup bias as well as our interpretation of prior research. Each author is based at a Canadian university, and has either earned a PhD in social psychology or is currently in the process of obtaining one. These experiences and positions allow for privileges that complicate or obstruct our understanding of issues related to intergroup prejudice or discrimination. Relatedly, a large percentage of the citations in this work come from papers where authors are White men from wealthy, industrialized North American or European institutions. As a result, the viewpoints represented here may not be appropriate for less wealthy and less resourced nations of non-White populations, and the low levels of diversity in our citations may overlook important frameworks that would have strengthened this work. Though we have tried to incorporate citations from non-Western contexts when possible, the citations still disproportionately promote White, Western perspectives and findings.

## Conclusion

Past studies have produced a large and diverse number of interventions that removed or lessened biases in judgment. Researchers interested in intergroup biases could benefit from understanding the promises and limitations of applying findings in the debiasing literature to the issue of discrimination. Recent work has already shown several cases of debiasing strategies being successfully adapted to reducing discrimination, though several prominent debiasing approaches remain unexplored or underexplored in the context of intergroup behavior. There are immense practical and theoretical benefits of turning to research on judgment biases to identify flexible, scalable, and durable interventions to reduce discrimination. Researchers owe this challenge a wide lens and open mind, as fruitful developments in this line of work are likely to come from a greater understanding of judgment biases in general.

## Notes

1. Specifically, of 96 total citations in 2022, Fischhoff (1982) was cited in Muhammad et al. (2022), Larrick (2004) was cited in Pedersen and Nielsen (2022), and Milkman et al. (2009) was cited in Jones et al. (2022).
